# Malacoplakia of the Uterine Cervix: A Case Report

**DOI:** 10.3390/pathogens10030343

**Published:** 2021-03-15

**Authors:** Adela Saco, Natalia Rakislova, Lorena Marimon, Aureli Torne, Berta Diaz-Feijoo, Rafael Salvador, Silvia Alos, Dercio Jordao, Juan Carlos Hurtado, Jaume Ordi

**Affiliations:** 1 Department of Pathology, Hospital Clinic, University of Barcelona, 08036 Barcelona, Spain; rakislova@clinic.cat (N.R.); lorena.marimon@isglobal.org (L.M.); salos@clinic.cat (S.A.); jordi@clinic.cat (J.O.); 2 Institut d’Investigacions Biomèdiques August Pi I Sunyer (IDIBAPS), 08036 Barcelona, Spain; atorne@clinic.cat (A.T.); bdiazfe@clinic.cat (B.D.-F.); 3 Institute of Global Health (ISGlobal), 08036 Barcelona, Spain; juancarlos.hurtado@isglobal.org; 4 Institute of Gynecology, Obstetrics and Neonatology, University of Barcelona, 08036 Barcelona, Spain; 5 Department of Radiology, Hospital Clinic, University of Barcelona, 08036 Barcelona, Spain; rsalvado@clinic.cat; 6 Department of Pathology, Hospital Central de Quelimane, Maputo, Mozambique; dercio.jordao@gmail.com; 7 Department of Microbiology, Hospital Clinic, University of Barcelona, 08036 Barcelona, Spain

**Keywords:** malacoplakia, uterine cervix, inflammatory pathology, Michaelis–Gutmann bodies

## Abstract

Malacoplakia is an uncommon chronic granulomatous inflammation that rarely affects the female genital tract. A case of a 78-year-old woman with malacoplakia involving the uterine cervix and the vagina is described. The patient complained of vaginal bleeding. Clinically, a 13-mm mass was detected in the cervix, which was confirmed by ultrasound scan and magnetic resonance imaging. Histological examination showed a dense histiocytic infiltrate with abundant Michaelis–Gutmann bodies involving the uterine cervix and the upper vagina. The presence of *Escherichia coli* was confirmed in the lesion by immunohistochemistry and polymerase chain reaction. Only 12 cases of cervical malacoplakia have been reported to date. This condition should be included in the differential diagnosis of cervical tumors.

## 1. Introduction

Malacoplakia is an uncommon chronic granulomatous condition that usually affects the urinary tract. It was first described by Michaelis et al. in 1902 [1]. The disease results from the inability of macrophages to destroy phagocytized bacteria and is usually associated with coliform infections, and particularly with *Escherichia coli*. Although malacoplakia is usually subsequent to infections, it has been described in association with malignant tumors [2].

Histologically, the lesion is characterized by the presence of large histiocytes, some of which contain pathognomonic Michaelis–Gutmann (MG) bodies. These structures are nucleus sized, basophilic bodies containing calcium, sometimes with a laminated structure and a bull’s-eye appearance. The histiocytic infiltrate is usually accompanied by a mixed inflammatory infiltrate composed of plasma cells and leukocytes.

Malacoplakia of the female genital tract is a rare disorder with less than 40 cases having been described to date [3,4,5,6,7,8,9,10,11,12,13,14,15,16,17,18,19], and only 12 cases of cervical involvement have been reported [5,6,7,9,10,14,15,16,17,18].

## 2. Case Report

A 78-year-old woman complained of vaginal bleeding that had persisted for one month that had not resulted in anemia (hemoglobin 122 g/L). The patient had a history of Sjögren’s syndrome treated with corticoids, a pulmonary thromboembolism 3 months before, and had cognitive deficit secondary to an episode of cerebral ischemia. The vaginal bleeding was confirmed by the caretakers.

**Figure 1:**
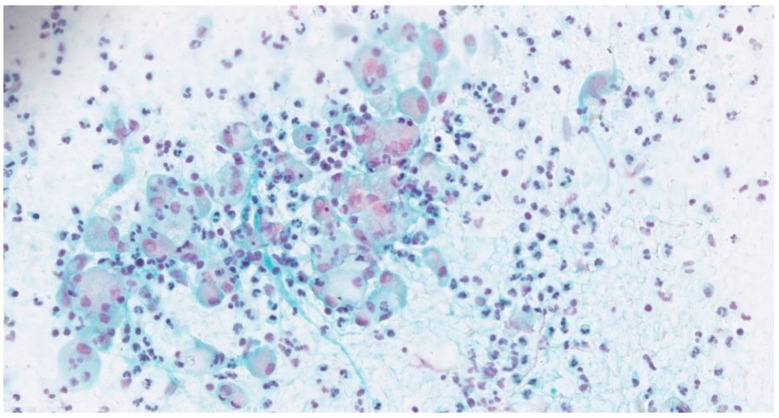
Cervicovaginal Pap-smear with abundant histiocytes and neutrophils (Papanicolaou stain, 400×).

**Figure 2:**
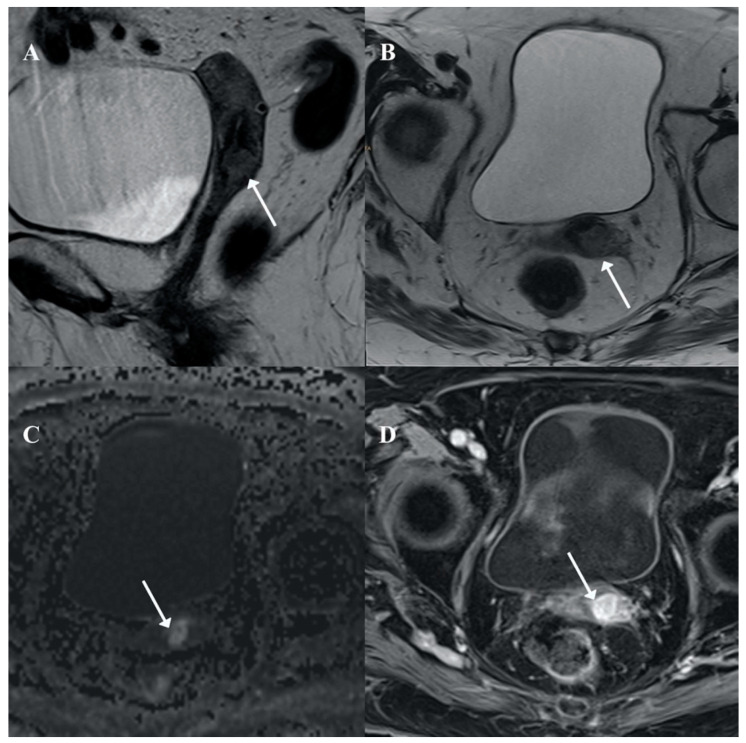
Pelvic magnetic resonance imaging (MRI). An infiltrative cervical mass (arrow) is seen in axial (**A**) and sagittal (**B**) T2 weighted MRI with stromal infiltration as a disruption of the cervical stroma hypointensity and focal infiltration of superior vaginal third (arrow in A). The lesion showed restricted diffusion in high b value (**C**: DWI b 800) and contrast enhancement (**D**: Post-contrast axial T1w fat saturated MRI).

The gynecological examination was limited due to pain. The ultrasound examination showed a 14 × 7-mm mass located in the posterior lip of the uterine cervix, with no other pathologic findings. A Pap-smear test and biopsy samples from the cervix and vagina were taken. The Pap-smear showed abundant large histiocytes and glandular cells with mild, non-specific atypia (Figure 1). The cervical and vaginal biopsies showed granulation tissue with a mixed inflammatory infiltrate and several histiocytes with granulated cytoplasm. No epithelial dysplasia was seen in any sample. Magnetic resonance imaging confirmed a 14-mm mass located in the cervix, showing stromal infiltration of the cervical stroma and focally involving the upper vagina (Figure 2). The patient did not receive any additional treatment. In spite of the absence of histological confirmation and due to the impossibility of conducting a proper clinical evaluation due to the pain, the lesion was clinically diagnosed as suspicious of cervical cancer, based on the imaging exams (ultrasound scan, magnetic resonance), the vaginal bleeding and the non-specific cytologic atypia. An informed consent was signed by the patient.

**Figure 3:**
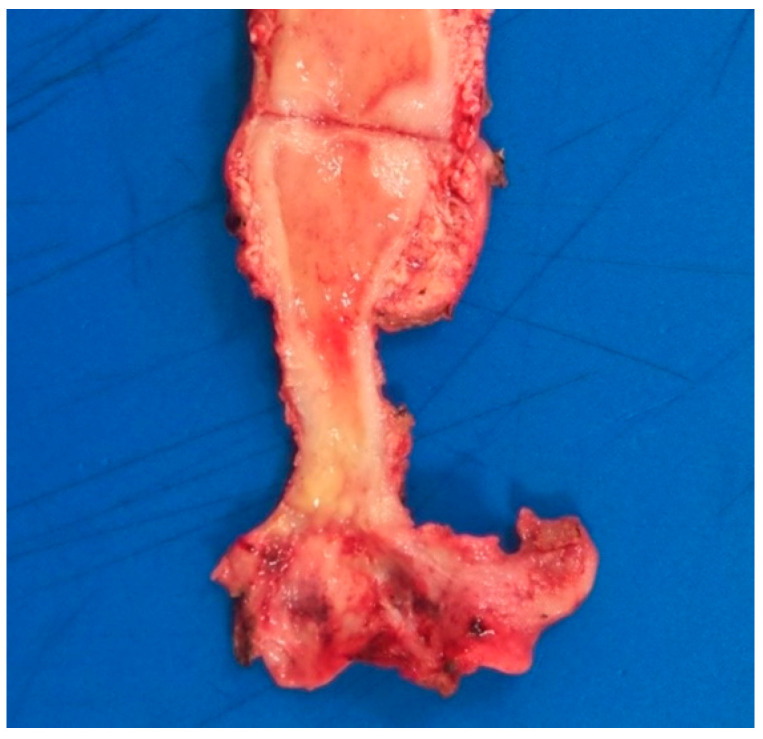
Macroscopic image showing a yellowish lesion with poorly defined margins involving the cervix and upper vagina.

A total hysterectomy was performed due to clinical suspicion of cervical cancer. Macroscopically, the cervix showed a yellowish and indurated lesion with poorly delimited margins, involving the whole uterine cervix and extending focally to the upper vagina (Figure 3).

The specimen was fixed in 10% neutral buffered formalin and embedded in paraffin following routine procedures. Paraffin sections were stained with hematoxylin and eosin, periodic acid-Schiff (PAS), and von Kossa. *E. coli* was detected using a mouse monoclonal antibody (Anti-E. coli LPS antibody 2D7/1 [ab35654], 1:100; Abcam, Cambridge, UK) following the manufacturer’s protocol. Immunohistochemistry was performed with the Autostainer Link 48 automated system (Dako Co., Carpinteria, CA, USA) using the EnVision system (Dako) and magenta as chromogen.

A polymerase chain reaction (PCR) for *E. coli* was performed in paraffin-embedded tissue. For DNA extraction, 10 µm thick sections of formalin-fixed, paraffin-embedded tissue were incubated overnight in 20 μL of proteinase K solution (1 mg/mL) at 56 °C. Subsequently, proteinase K was heat inactivated by incubation of the sections at 95 °C for 10 min, and samples were spun and cooled down at −20 °C for 1–2 min. DNA was isolated using a commercially available kit (QIAamp Tissue Kit; Qiagen, Hilden, Germany). DNA yields were quantified spectrophotometrically using the NanoDrop ND–1000 (Thermo Scientific NanoDrop, Wilmington, DE, USA). Detection of *E. coli* was performed using specific primers and probes (LightMix^®^ Modular *E. coli* uidA, TIB MOLBIOL Syntheselabor GmbH, Berlin, Germany). The LightMix^®^ modular assay was run in a LightCycler^®^ 480 II instrument (Roche Diagnostics, Indianapolis, IN, USA). PCR cycle threshold values > 37 were considered negative.

**Figure 4:**
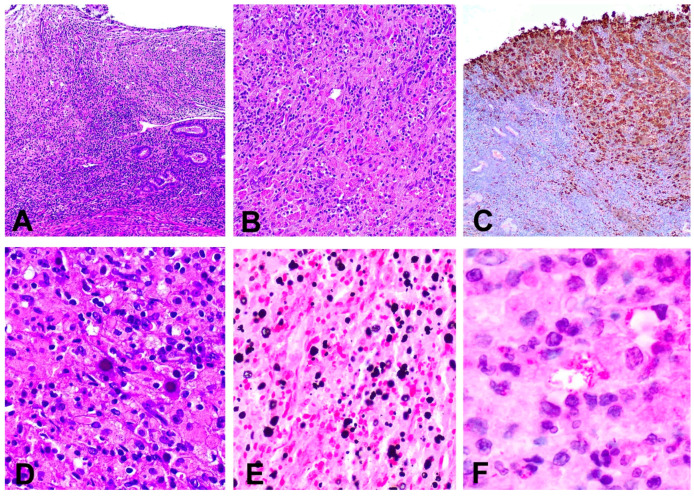
(**A**) Inflammatory infiltrate replacing the lamina propria and ulcerating the superficial epithelium (hematoxylin and eosin, 40×); (**B**) Histiocytic infiltrate in the cervical stroma (hematoxylin and eosin, 200×); (**C**) Immunohistochemical stain for CD68 showing a dense infiltrate composed by macrophages (immunohistochemical stain anti CD68); (**D**) Abundant Michaelis–Gutmann bodies (hematoxylin ad eosin 200×); (**E**) Presence of intra and extracytoplasmic Michaelis–Gutmann bodies positive for von Kossa stain (100×); (**F**) Escherichia coli bacilli present inside the cytoplasm of histiocytes (immunohistochemical stain anti *E. coli*, 600×).

Microscopically, a dense inflammatory was identified in the cervix and upper vagina. The infiltrate involved the lamina propria and extensive ulceration of the superficial epithelium was observed and was composed mostly by CD68 positive macrophages with a large foamy or granular cytoplasm showing abundant basophilic inclusions in the cytoplasm. The surgical margins were free of infiltrate. These inclusions were laminated and positive with PAS and von Kossa stains (MG bodies). Immunohistochemically, abundant intracytoplasmic *E. coli* bacilli were identified (Figure 4). Additionally, the PCR detected *E. coli* DNA, confirming the diagnosis. 

The patient was asymptomatic 13 months after surgery.

## 3. Discussion 

Malacoplakia is an uncommon inflammatory process which usually involves the genitourinary tract. The presentation as a primary lesion in the female genital tract is rare, and less than 40 cases have been reported to date [3,4,5,6,7,8,9,10,11,12,13,14,15,16,17,18,19]. The most common site of involvement is the vagina [14], and only 13 cases of cervical malacoplakia have been reported, including the present case [5,6,7,9,10,14,15,16,17,18] (Table 1). The age of the patients at diagnosis ranged from 27 to 83 years, with a mean of 66 years. In addition to the uterine cervix, other anatomic sites were involved in seven patients (endometrium in three cases, vagina in three cases, and pelvic wall in one case) [5,6,10,14,15,18]. The most common presenting symptom was vaginal bleeding (present in 83% of the patients), but other clinical findings, such as cervical mass, vaginal discharge, cervical ulceration, abdominal pain or friable cervix have been reported. Five patients presented acquired immunosuppression: two secondary to HIV infection and three associated with corticoid therapy [5,9,16].

The diagnosis is based on microscopic findings of macrophages with MG bodies, which can occasionally be identified in Pap smears [5,6,16,17]. However, MG bodies can be abundant or scant and may be completely absent in the cytological samples, as in the present case. These structures can be identified inside or outside the cytoplasm of macrophages and typically show positive staining with PAS and von Kossa stains [5,16,17]. In the present case, *E. coli*, the microorganism most commonly identified as causing malacoplakia, was detected immunohistochemically and by PCR in the lesion, confirming the diagnosis. This is the first malacoplakia of the uterine cervix in which the responsible microorganism was identified by immunohistochemistry. In the previously reported cases, *E. coli* infection was confirmed in two patients by tissue and urine culture [14,18]. In two other patients, a concomitant granuloma inguinale was present, and other agents, such as Klebsiella, have also been associated with malacoplakia [9].

Due to the few cases reported, the clinical management remains unclear. In the present case, the decision of performing a hysterectomy in the absence of a histological diagnosis was based on the evidence of a cervical mass in the imaging exams (US scan, MRI), together with the serious limitations to the clinical evaluation and her advanced age. It was thus considered that the advantages of solving the clinical symptoms and excluding/confirming a malignant tumor exceeded the risks of possible overtreatment. In this respect, it is interesting to note that, as clearly shown in Table 1, hysterectomy [6,7,14,18] is the most frequent treatment reported. Antibiotic therapy has also been reported in some patients [15,16,17]. All cases have shown a benign behavior. However, due to the limited experience, conclusions on the effectiveness of the different treatments cannot be drawn.

## 4. Conclusions

In summary, malacoplakia is an inflammatory alteration that can mimic or can be associated with a malignant tumor; in light of this, it is important remember that malacoplakia of female genital tract exists.

## Figures and Tables

**Table 1:** Reported cases of malacoplakia of the uterine cervix.

Reference	Age	Clinical Presentation	Other Structure Involved	Immune Suppression	Treatment	*E. coli*
[14]	64	Vaginal bleeding	Vagina	No	Hysterectomy	Urine culture
[16]	72	Vaginal bleeding. Cervical mass	No	Corticoid therapy	Died before treatment	No
[16]	66	Vaginal discharge. Friable cervix	No	No	Antibiotic	No
[6]	71	Vaginal bleeding. Abdominal pain. Cervical mass	Endometrium	No	Hysterectomy	No
[18]	83	Vaginal bleeding. Cervical mass	Pelvic wall	No	Hysterectomy	Tissue culture
[17]	78	Vaginal discharge. Friable cervix	No	No	Antibiotics	No
[5]	60	Vaginal bleeding. Cervical mass	Endometrium	Corticoid therapy	Not reported	No
[15]	74	Vaginal bleeding. Cervical mass	Endometrium	No	Antibiotics	No
[7]	69	Ulcerated cervix	No	No	Hysterectomy	No
[9]	27	Vaginal bleeding. Ulcerated cervix	No	AIDS	Died before treatment	No (GI)
[9]	36	Vaginal bleeding. Ulcerated cervix	No	AIDS	No follow-up	No (GI)
[10]	81	Vaginal bleeding. Cervical mass	Vagina	No	Not reported	No
**Present case**	78	Vaginal bleeding. Cervical mass. Abdominal pain	Vagina	Corticoid therapy	Hysterectomy	PCR

AIDS: Acquired immune deficiency syndrome; GI: granuloma inguinale; PCR: polymerase chain reaction.

## Data Availability

Data sharing not applicable.
